# Prognostic Significance of Frailty in Liver Cirrhosis Patients: A Prospective Single-Center Study

**DOI:** 10.3390/jcm15051943

**Published:** 2026-03-04

**Authors:** Maral Martin Mıldanoğlu, Atilla Akpınar, Koray Koçhan, Ahmet Bilici, Elmas Biberci Keskin, Hakan Şentürk

**Affiliations:** 1 Department of Medical Oncology, Faculty of Medicine, Istanbul Medipol University, 34214 Istanbul, Turkey; ahmetknower@yahoo.com; 2 Department of Gastroentrology, Cerrahpasa Faculty of Medicine, Istanbul University-Cerrahpasa, 34098 Istanbul, Turkey; atilla.akpinar@hotmail.com; 3 Department of Gastroentrology, Faculty of Medicine, Biruni University, 34295 Istanbul, Turkey; kochankoray@yahoo.com; 4 Department of Internal Medicine, University of Health Sciences, Sisli Hamidiye Etfal Training and Research Hospital, 34396 Istanbul, Turkey; 5 Department of Gastroentrology, Faculty of Medicine, Bezmialem Foundation University Medical Faculty Hospital, 34093 Istanbul, Turkey; drhakansenturk@yahoo.com

**Keywords:** frailty, cirrhosis, FFI, prognosis, mortality

## Abstract

**Background:** Liver cirrhosis is a systemic disease characterized by progressive hepatic dysfunction and frequent decompensation events. Conventional prognostic models such as the Child–Turcotte–Pugh (CTP) and Model for End-stage Liver Disease (MELD) scores primarily reflect liver-specific severity and may not fully capture the multidimensional vulnerability of patients with cirrhosis. Frailty, a syndrome reflecting reduced physiological reserve, has emerged as a potential prognostic marker in this population. **Methods:** In this prospective single-center cohort study, 134 patients with liver cirrhosis were enrolled between March and October 2021 and followed at three-month intervals. Frailty was assessed at baseline using the Fried Frailty Index (FFI). Patients were categorized as fit/prefrail or frail. The primary endpoints were cirrhosis-related complications, unplanned hospitalizations, and all-cause mortality. Associations between frailty, its individual components, and clinical outcomes were evaluated. **Results:** Frailty was present in 41% of patients. Frail patients were older and had higher MELD and CTP scores. During follow-up, frailty was significantly associated with higher rates of ascites (*p* < 0.001), hepatic encephalopathy (*p* < 0.001), hepatorenal syndrome (*p* < 0.001), spontaneous bacterial peritonitis (*p* = 0.01), and unplanned hospitalizations (*p* < 0.001). Mortality occurred in 22% of frail patients compared with 3.8% in non-frail patients (*p* < 0.001). Each frailty component, including reduced grip strength, slow gait speed, low physical activity, exhaustion, and unintentional weight loss, was independently associated with adverse outcomes. **Conclusions:** Frailty, as assessed by the Fried Frailty Index, is a strong predictor of complications, hospitalization, and mortality in patients with liver cirrhosis. Incorporating frailty assessment into routine clinical practice may improve risk stratification and guide long-term management strategies.

## 1. Introduction

Liver cirrhosis represents the advanced stage of chronic liver disease and is characterized by progressive parenchymal fibrosis and the disruption of normal liver architecture, leading to the formation of abnormal nodular structures. It is a chronic, and progressive condition, presenting with features of hepatocellular insufficiency and portal hypertension [1]. Despite advances in medical management, patients with cirrhosis remain at high risk for recurrent decompensation events, frequent hospitalizations, and premature death. Conventional prognostic models such as the Child–Turcotte–Pugh (CTP) and the Model for End-stage Liver Disease (MELD) are widely used to guide clinical decision-making. However, these scores primarily rely on liver specific laboratory and clinical parameters and may not adequately reflect the broader physiological vulnerability of patients with cirrhosis [2].

Frailty is a clinical syndrome resulting from cumulative decline across multiple physiological systems, leading to increased vulnerability to adverse outcomes and reduced resilience to stressors [3]. Although it shares overlapping features with sarcopenia, frailty does not represent the same condition. In general, sarcopenia refers to the loss of muscle mass and may represent one component of frailty; however, the measurement of muscle strength alone is insufficient to define frailty [4]. Frailty can be assessed using various scales or scoring systems. In clinical practice, the most commonly applied tool is the Fried Frailty Index (FFI), although alternative measures such as the Clinical Frailty Scale (CFS) and the Liver Frailty Index (LFI) are also utilized [5,6,7]. Over the past decade, frailty has been associated with mortality in patients with liver cirrhosis, as well as with clinically relevant events including hepatic encephalopathy, ascites development, decompensation, and hospitalizations. A summary of key studies evaluating frailty in cirrhosis is provided Table 1.

In our study, we investigated whether frailty in patients with cirrhosis is associated with major complications such as ascites, hepatic encephalopathy, variceal bleeding, and hepatorenal syndrome, and whether it constitutes a risk factor for unplanned hospitalization and mortality.

## 2. Methods

### 2.1. Study Design and Participants

**Figure 1:**
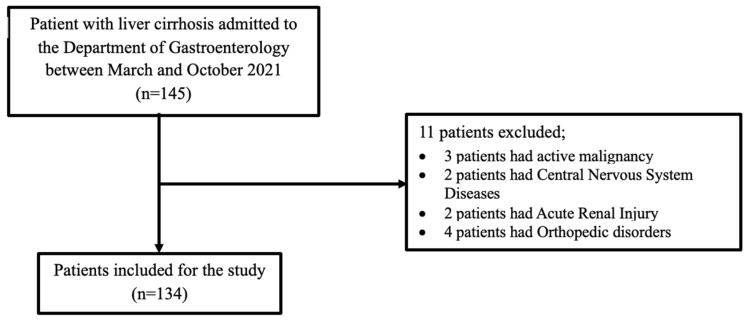
Flow diagram of the study population.

This prospective single-center cohort study enrolled patients aged 18–80 years who were admitted to the Department of Gastroenterology at Bezmialem Foundation University Medical Faculty Hospital, Istanbul. with a diagnosis of liver cirrhosis. The diagnosis of liver cirrhosis was established based on a combination of clinical findings, laboratory parameters, and radiologic imaging results (such as ultrasonography, computed tomography, or magnetic resonance imaging showing nodular liver surface, splenomegaly, or portal hypertension features). Exclusion criteria were the presence of active malignancy, central nervous system disease, acute kidney injury, overt hepatic encephalopathy, or any orthopedic condition that limited mobility. Inclusion and exclusion criteria of the study demonstrated in Table 2. From March to October 2021, 145 patients with liver cirrhosis were evaluated for participation. Eleven patients were excluded for not meeting the eligibility criteria, resulting in a final cohort of 134 patients. The specific reasons for exclusion are illustrated in Figure 1. Included patients were followed at 3-month intervals through outpatient clinic visits or telephone interviews. During follow-up, the development of cirrhosis-related complications and the occurrence of unplanned hospitalizations were systematically assessed. The study endpoints were defined as the occurrence of cirrhosis-related complications, unplanned hospitalizations, and death at any time after enrollment.

The study was approved by the Bezmialem Vakıf University Health Practice and Research Hospital Directorate, with ethics committee approval dated 8 September 2020 (decision no. 15/310). Written informed consent was obtained from all patients or their designated legal representatives.

### 2.2. Data Collection

At the baseline visit, the following data were recorded for each patient: age, sex, time since the initial diagnosis of cirrhosis, body-mass index (BMI), comorbidities such as hypertension, diabetes mellitus, and coronary artery disease; etiology of cirrhosis; Child–Turcotte–Pugh (CTP) and Model for End-stage Liver Disease (MELD) scores; presence of varices and ascites; and current use of diuretics, beta-blockers, and rifaximin. Following the collection of these baseline data, patient frailty was assessed using the Fried Frailty Index (FFI). During FFI evaluation, data on parameters such as unintentional weight loss, reduced physical activity, and self-reported exhaustion were obtained from patients and their caregivers. Muscle strength was assessed using handgrip strength, with cut-off values defined according to body mass index (BMI). Walking speed was evaluated using the 4.5 m gait test. Patients meeting three or more of these five criteria were classified as frail, those meeting one or two criteria as prefrail, and those meeting none of the criteria as fit. (Table 3).

### 2.3. Statystical Analysis

Based on previous studies investigating the prevalence of frailty in patients with cirrhosis, the minimum required sample size was calculated as 176 subjects to detect a 15% difference in Fried criteria scores between groups (20% vs. 5%) with a 95% confidence interval and 80% power [13].

Prospectively collected data were recorded in Microsoft Excel throughout the study and subsequently analyzed using R software (version 4.1.2; R Foundation for Statistical Computing, Vienna, Austria; available at: http://www.r-project.org/). Statistical analyses employed the following packages: gtsummary v1.5.2, survival v3.2-13, survminer v0.4.9, and pROC v1.18.0. The normality of continuous variables was assessed using the Kolmogorov–Smirnov and Shapiro–Wilk tests, as well as Q–Q plots and histograms. Continuous variables were expressed as median (25th–75th percentile) and mean ± standard deviation, while categorical variables were summarized as frequencies (percentages). Group comparisons were performed using the Mann–Whitney U test for continuous data. For categorical variables, Pearson’s chi-square test was applied when cell counts were sufficient, and Fisher’s exact test was used otherwise. The discriminatory ability of selected parameters for predicting clinical outcomes was evaluated using receiver operating characteristic (ROC) curve analysis.

## 3. Results

### 3.1. Patient Characteristics

A total of 134 patients (69 women [51.5%] and 65 men [48.5%]) were enrolled in the study. The mean age of the overall cohort was 62 years (range: 24–80). These patients were divided into two groups: robust (n = 39, 29%) or prefrail (n = 40, 30%) and frail (n = 55, 41%).

When baseline characteristics were compared, the frail patient group was older (*p* < 0.001). Hypertension (*p* = 0.028), diabetes mellitus (*p* = 0.033), and coronary artery disease (*p* < 0.001) were more frequent in the frail group. MELD and CTP scores were also significantly higher among frail patients (*p* < 0.001). In contrast, cirrhosis etiology, presence of varices, history of variceal bleeding, and the proportion of patients receiving L-ornithine L-aspartate were similar between the two groups. Baseline characteristics of the two groups are shown in Table 4.

### 3.2. Complications, Hospitalizations and Mortality in Patient Groups

In the comparative analysis of the two groups, the frail cohort demonstrated a significantly higher frequency of ascites, hepatic encephalopathy, and hepatorenal syndrome (*p* < 0.001). Data regarding the comparisons of complications and outcomes are presented in Table 5.

**Figure 2:**
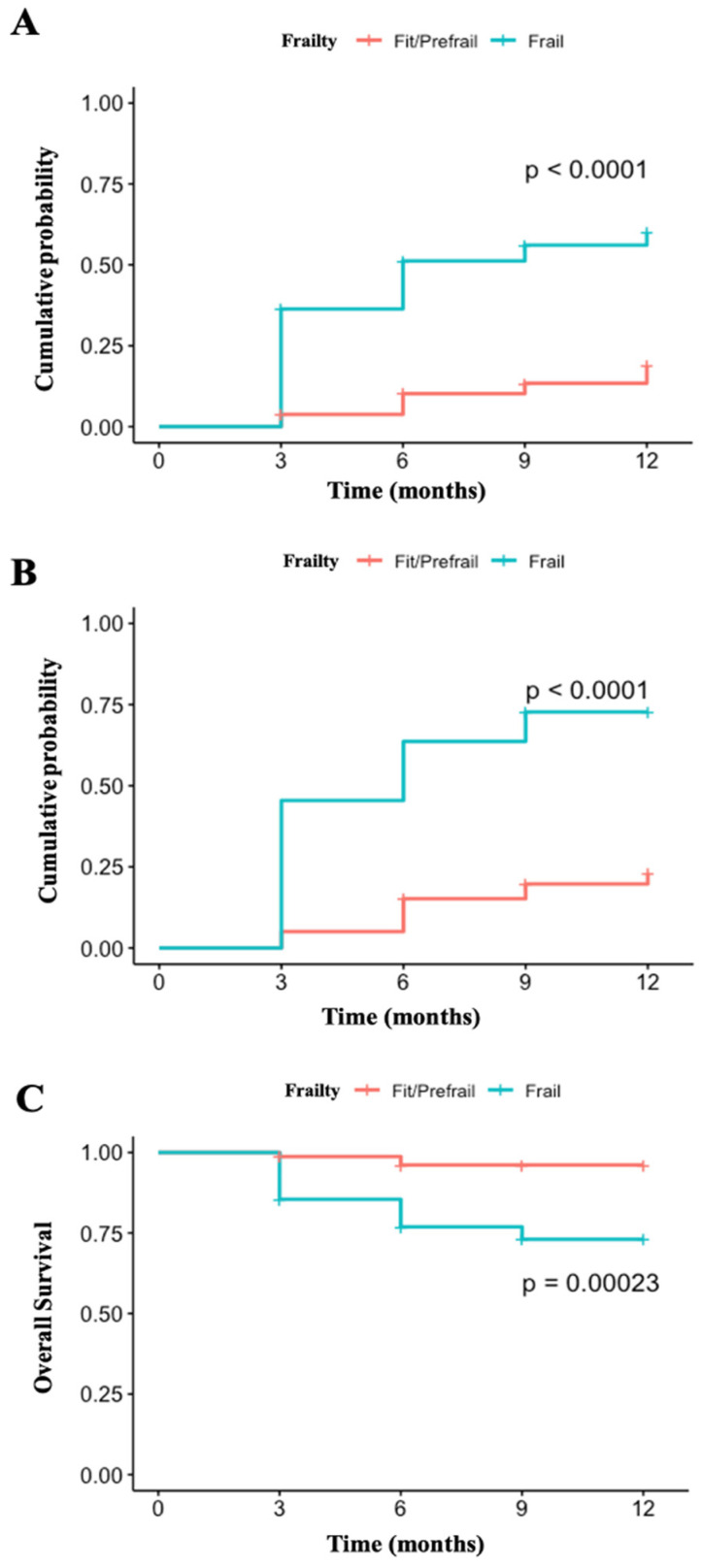
Comparison of clinical outcomes between frail and prefrail/robust cohorts. (**A**): Comparison of cumulative probabilities of unplanned hospitalizations between the two groups; (**B**): Comparison of the two groups with respect to all adverse outcomes; (**C**): Comparison of overall survival between the two groups.

When unplanned hospitalizations, mortality, and all adverse endpoints were assessed collectively, the frail patient group demonstrated significantly worse outcomes (*p* < 0.001). Notably, both unplanned hospitalizations and mortality were individually more frequent among frail patients (*p* < 0.001). Graphical representations of all adverse endpoints, unplanned hospitalizations, and survival are provided in Figure 2.

### 3.3. Predictive Performance of Frailty Versus Conventional Scoring Systems

**Figure 3:**
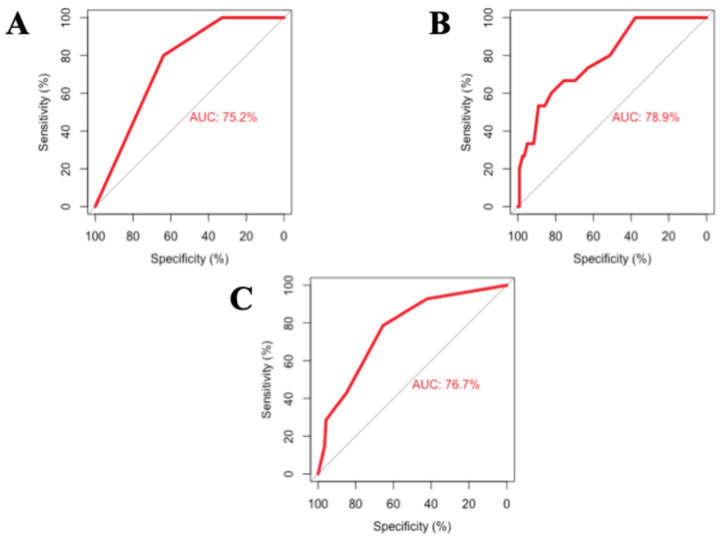
ROC curve analyses illustrating the predictive performance for mortality: (**A**): Frailty (**B**): MELD score and (**C**): CTP score.

The predictive performance of the frailty scale for mortality was compared with the CTP and MELD scores using ROC curve analysis. The MELD score showed the highest discriminative power (AUC: 78.9%), followed by the CTP score (AUC: 76.7%) and the frailty scale (AUC: 75.2%). Although the MELD and CTP scores exhibited slightly higher AUC values, the frailty scale demonstrated a comparable and significant predictive capacity for mortality in patients with liver cirrhosis. The ROC analysis of these three parameters regarding mortality prediction is illustrated in Figure 3.

**Figure 4:**
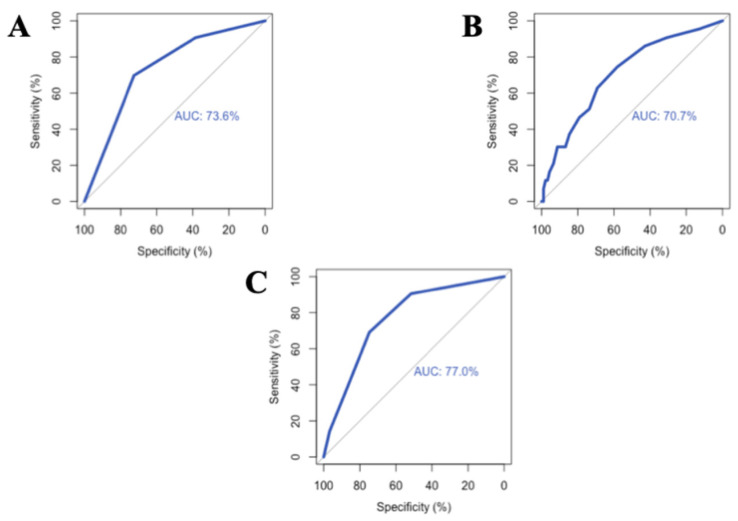
ROC curve analyses illustrating the predictive performance for unplanned hospitalizations: (**A**): Frailty (**B**): MELD score and (**C**): CTP score.

The predictive performance of the frailty for unplanned hospitalizations was evaluated and compared with the CTP and MELD scores using ROC curve analysis. For this outcome, the CTP score showed the highest discriminative power (AUC: 77.0%), followed by the frailty scale (AUC: 73.6%) and the MELD score (AUC: 70.7%). These results indicate that the frailty scale is a significant and reliable predictor of unplanned hospitalizations, performing comparably to traditional clinical scoring systems. The ROC analysis of these three parameters regarding the prediction of unplanned hospital admissions is illustrated in Figure 4.

### 3.4. Relationship Between Frailty Parameters and Clinical Complications

Among the 134 patients included in the study, 20 (14.9%) had unintentional weight loss, whereas 114 (85.1%) did not. When these two groups were compared, the development of ascites (*p* = 0.004), hepatic encephalopathy (*p* = 0.02), and hepatorenal syndrome (*p* = 0.037) was more frequent in patients with unintentional weight loss. In addition, unplanned hospitalizations were significantly more common in this group (*p* < 0.001). No significant difference in mortality was observed between the two groups.

Among the 134 patients included in the study, 83 (61.9%) had reduced muscle strength, while 51 (38.1%) had normal muscle strength. In the group with reduced muscle strength, the development of ascites, hepatic encephalopathy, and unplanned hospitalizations was significantly more frequent (*p* < 0.001). Furthermore, mortality was markedly higher in patients with reduced muscle strength compared with those with normal strength (17% vs. 2%, *p* = 0.008).

Among the 134 patients, 66 (49.3%) had reduced gait speed, whereas 68 (50.7%) had normal gait speed. The incidence of ascites (*p* = 0.007), hepatic encephalopathy (*p* < 0.001), unplanned hospitalizations (*p* < 0.001), and mortality (*p* = 0.048) was significantly higher in the group with reduced gait speed.

Among the study population, 51 patients (38.1%) were classified as having low physical activity, while 83 patients (61.9%) had normal physical activity levels. Patients with reduced physical activity exhibited significantly higher rates of ascites, hepatic encephalopathy, hepatorenal syndrome and unplanned hospitalizations (*p* < 0.001). Moreover, mortality was also significantly increased in this group compared with those with normal activity levels (*p* = 0.015).

Exhaustion was present in 49 patients, whereas 85 patients did not exhibit this clinical condition. In the exhaustion group, the incidence of ascites, hepatic encephalopathy, hepatorenal syndrome, and unplanned hospitalizations was significantly higher (*p* < 0.001). In addition, the occurrence of spontaneous bacterial peritonitis was also more frequent in this group (*p* = 0.006). Mortality rates were likewise higher in the group with exhaustion (*p* = 0.01).

## 4. Discussion

This study demonstrates that frailty, assessed by the Fried Frailty Index (FFI), is a strong predictor of adverse outcomes in patients with cirrhosis (*p* < 0.001). Over the course of one year, frailty was consistently associated with higher risks of decompensation, unplanned hospitalizations, and mortality (*p* < 0.001). Notably, these associations persisted even when traditional prognostic tools such as the CTP and MELD scores were taken into account, suggesting that frailty captures aspects of patient vulnerability that are not reflected by liver-specific severity indices. By highlighting the prognostic significance of frailty, our findings support the growing recognition that cirrhosis is not solely a hepatic disease but a systemic condition in which physical resilience and functional reserve play a pivotal role in patient outcomes.

Previously, Lai et al. and Xu et al. demonstrated that frailty is independently associated with increased waitlist mortality among liver transplant candidates, highlighting the prognostic importance of frailty in the transplant setting, while Tandon et al. confirmed this association in an outpatient cohort not restricted to transplant candidates [6,7,14]. Furthermore, Tapper et al. reported that frailty was strongly associated with mortality among hospitalized patients with cirrhosis [15]. In our study, a total of 15 deaths occurred during follow-up: 12 in frail patients (21.8%), 3 in prefrail patients (7.5%), and none in fit patients (0%). Cumulative survival analysis revealed that mortality was significantly higher in frail patients compared with non-frail patients (*p* < 0.001), consistent with the existing literature.

Previously, Dunn et al. demonstrated that cirrhotic patients on the transplant waitlist who were identified as frail based on gait speed experienced more hospitalizations due to cirrhosis-related complications [11]. Tandon et al. and Roman et al. evaluated an outpatient cirrhosis cohort, not restricted to transplant candidates, and similarly found frailty to be an independent predictor of unplanned hospitalizations [5,6]. In our study, a total of 43 unplanned hospitalizations were observed: 30 occurred in frail patients (54.5%), 9 in prefrail patients (22.5%), and 4 in fit patients (10.3%). Cumulative analysis demonstrated that the incidence of unplanned hospitalizations was significantly higher in frail patients compared with non-frail patients (*p* < 0.001), consistent with findings from the existing literature.

Frailty was found to be independently associated with the development of ascites during follow-up (*p* < 0.001), indicating a direct relationship with this complication. In contrast, most previous studies have assessed ascites only within composite decompensation outcomes and have described frailty primarily as a reduction in overall physiological reserve that increases vulnerability to complications, rather than as a factor directly related to a single event [4,16]. Our findings therefore expand the literature by suggesting that frailty may have particular relevance for the development of ascites in cirrhosis.

In our study, frailty was independently associated with the development of hepatic encephalopathy (HE) during follow-up (*p* < 0.001). While many previous studies have considered frailty mainly as a global reduction in physiological reserve predisposing patients to complications in general, only a limited number of studies have specifically examined its relationship with HE. Among these, the combination of the Clinical Frailty Scale (CFS) and the Montreal Cognitive Assessment (MoCA) has been shown to predict the development of HE [17]. Our findings complement this limited evidence by demonstrating that frailty measured with the Fried Frailty Index (FFI) is also associated with HE, suggesting that different frailty instruments consistently identify patients at increased risk for this complication.

Our study also showed that frailty was associated with the development of HRS (*p* < 0.001) and SBP (*p* = 0.01). Unlike ascites and hepatic encephalopathy, which have been more frequently examined in relation to frailty, there are no studies that have specifically evaluated HRS and SBP as individual outcomes. In the existing literature, these complications have generally been assessed only as part of composite decompensation endpoints rather than analyzed separately [11,16]. By demonstrating an association between frailty and the occurrence of both HRS and SBP, our findings contribute novel evidence to this underexplored area and suggest that frailty assessment may provide useful information for anticipating these serious complications of cirrhosis.

Roman et al. previously demonstrated that frail cirrhotic patients experienced composite adverse outcomes (including hospitalization, falls, and death) more frequently than non-frail patients [5]. In our study, we similarly investigated the relationship between frailty and adverse outcomes; however, unlike prior work, we defined composite outcomes as major complications of cirrhosis (ascites, HE, HRS, SBP, variceal bleeding, and development of HCC), unplanned hospitalizations, and mortality. During follow-up, 57 patients experienced adverse outcomes: 40 in the frail group (72.7%), 13 in the prefrail group (32.5%), and 4 in the fit group (10.3%). Adverse events were variceal bleeding, infection-related hospitalization, and development of significant ascites in fit patients. Of note, adverse outcomes in frail patients clustered particularly in the first 3 months of follow-up. Cumulative analysis demonstrated that adverse outcomes were significantly more frequent in frail patients compared with non-frail patients (*p* < 0.001). The ability of the Fried Frailty Index (FFI) to predict adverse outcomes in our cohort can be explained by the fact that frailty is not merely a marker of physical weakness, but a clinical manifestation of systemic physiological exhaustion. In liver cirrhosis, the transition from compensated to decompensated states is often driven by a complex inflammatory milieu and significant immune cell dysfunctions [18]. While conventional scores like MELD and CTP are essential for assessing organ-specific failure, they may fall short in capturing the ‘multidimensional vulnerability’ caused by this systemic decline. This vulnerability is particularly critical in the context of Acute-on-Chronic Liver Failure (ACLF), where underlying immune exhaustion predisposes patients to rapid clinical deterioration following minor insults [19]. Our results suggest that the FFI captures this systemic fragility, serving as a clinical surrogate for the patient’s overall biological reserve and their susceptibility to acute deteriorations.

To account for variable follow-up duration, incidence rates were calculated per 100 person-months. The cumulative follow-up time was 564 person-months in the frail group and 900 person-months in the non-frail group. The incidence of ascites was substantially higher in frail patients compared with non-frail patients (3.37 vs. 0.78 per 100 person-months, *p* < 0.001). Similarly, hepatic encephalopathy occurred more frequently in the frail group (4.79 vs. 0.44 per 100 person-months, *p* < 0.001). The incidence of HRS was also markedly increased among frail patients (2.30 vs. 0.11 per 100 person-months, *p* < 0.001). SBP occurred only in the frail group (0.89 vs. 0 per 100 person-months, *p* < 0.001). Unplanned hospitalizations were significantly more common in frail patients (5.32 vs. 1.44 per 100 person-months, *p* < 0.001). Mortality was likewise higher in the frail group (2.13 vs. 0.33 per 100 person-months, *p* < 0.001). Finally, the composite outcome showed a markedly elevated incidence among frail patients compared with non-frail patients (7.09 vs. 1.89 per 100 person-months, *p* < 0.001).

The prevalence of reduced grip strength in patients with cirrhosis has been reported to range between 38% and 62% [20,21]. Miwa et al. have demonstrated that impaired grip strength is associated with the development of covert hepatic encephalopathy (CHE) [21]. Moreover, reduced grip strength has been shown to predict increased mortality in cirrhotic populations [22,23]. In our cohort of 134 patients, reduced grip strength was identified in 83 (62%). When patients compared, the development of hepatic encephalopathy was significantly more frequent in those with reduced grip strength (*p* < 0.001), consistent with earlier studies. In addition to these previously reported associations, our study further demonstrated that ascites and hepatorenal syndrome (HRS) occurred more frequently in patients with reduced grip strength (*p* = 0.002). Furthermore, low grip strength was significantly associated with unplanned hospitalizations (*p* < 0.001) and higher mortality rates (*p* = 0.008).

Unintentional weight loss has been reported in 8–54% of patients with cirrhosis in previous studies [5,20]. Among liver transplant candidates, unintentional weight loss has been identified as a significant predictor of mortality [14]. In our cohort, unintentional weight loss was observed in 20 patients (15%). Patients with weight loss experienced significantly higher rates of ascites (*p* = 0.004), HE (*p* = 0.02), HRS (*p* = 0.037) and unplanned hospitalizations (*p* < 0.001) compared with those without weight loss.

In the literature, reduced gait speed has been reported in 26% of patients with cirrhosis [5,20]. Reduced gait speed has also been associated with increased mortality in cirrhotic patients in different studies [24,25]. In our cohort, reduced gait speed was observed in 66 patients (49%). Patients with reduced gait speed had higher rates of ascites (*p* = 0.007), HE (*p* < 0.001), and HRS (*p* = 0.004). Consistent with previous studies, reduced gait speed in our cohort was also significantly associated with unplanned hospitalizations (*p* < 0.001) and higher mortality (*p* = 0.048).

In previous studies, low physical activity has been identified as a frailty parameter in 32–34% of patients with cirrhosis [5,20]. In our cohort, reduced physical activity was observed in 51 patients (38%). Patients with low physical activity had significantly higher rates of ascites (*p* < 0.001), HE (*p* < 0.001), HRS (*p* < 0.001), and SBP (*p* = 0.037) compared with those with preserved activity levels. Furthermore, reduced physical activity was associated with more frequent unplanned hospitalizations (*p* < 0.001 and higher mortality (*p* = 0.015).

In previous studies, exhaustion has been identified as a frailty parameter in 20% to 62% of patients with cirrhosis [5,20]. In our cohort, exhaustion was reported in 49 patients (36.5%). Patients with exhaustion had significantly higher rates of ascites (*p* < 0.001), HE (*p* < 0.001), HRS (*p* < 0.001), SBP (*p* = 0.006), as well as increased unplanned hospitalizations (*p* < 0.001) and mortality (*p* = 0.01).

The first limitation of our study is that the number of patients did not reach the sample size estimated in the initial power analysis. Although 176 patients were planned, only 134 could be enrolled between March 2021 and October 2021, largely due to reduced admissions during the COVID-19 pandemic. The second limitation concerns the follow-up period. Although all patients were initially intended to be followed for one year, by the data cut-off date in May 2022, only 100 patients had completed a full year of follow-up, while 20 patients had 9 months and 14 patients had 6 months of follow-up.

Compared with previous studies, our work is distinctive in that frailty and its subcomponents were assessed using the more objective and detailed Fried frailty criteria and evaluated in a randomized cohort of all cirrhotic patients presenting to the gastroenterology clinic (including outpatients, inpatients, and transplant candidates) with respect to all major complications of cirrhosis, unplanned hospitalizations, mortality, and composite outcomes.

## 5. Conclusions

In our study, frailty and all of its subcomponents were each identified as independent predictors of ascites, hepatic encephalopathy, unplanned hospitalizations, and mortality. Moreover, they also predicted the composite adverse outcome defined as the combined occurrence of major complications, hospitalizations, and mortality. Although the target sample size and follow-up duration could not be fully achieved, the predictive ability demonstrated by each parameter individually as well as collectively was striking. Based on these findings, the assessment of frailty, particularly using the Fried Frailty Index (FFI), may be recommended as a valuable tool for the long-term follow-up and risk stratification of patients with cirrhosis.

## Figures and Tables

**Table 1:** Summary of Studies Assessing Frailty in Cirrhosis.

Study	Frailty Measure	Patient Population	Main Findings
Roman, E. 2021 [5]	FFI	135 outpatients with cirrhosis, 135 controls	Frail patients had higher rates of ascites, mortality, and hospitalizations, with longer hospital stays.
Xu, C.Q. 2021 [7]	LFI	247 cirrhotic patients on the liver transplant waiting list	Frailty was associated with increased mortality during the waiting period.
Haugen, C.E. 2020 [8]	LFI	882 patients evaluated for liver transplantation	Frail patients had a twofold higher mortality while on the waiting list.
Lai, J.C. 2019 [9]	LFI	1044 patients on the transplant waiting list	Frailty was associated with higher mortality.
Lai, J.C. 2017 [10]	Physical frailty parameters	536 cirrhotic patients	Low physical activity, slow walking speed, and reduced grip strength were associated with higher mortality on the waiting list.
Dunn, M.A. 2016 [11]	Grip strength and walking speed	373 cirrhotic patients	Reduced walking speed was linked to cirrhosis-related complications, more hospitalizations, and longer length of stay.
Cron, D.C. 2016 [12]	FFI	500 cirrhotic patients evaluated for liver transplantation	Frailty associated with higher rates of hepatic encephalopathy
Tandon, P. 2016 [6]	CFS	300 outpatients with cirrhosis	Frailty was associated with unplanned hospitalizations and increased mortality risk.

FFI: Fried Frailty Index, LFI: Liver Frailty Index, CFS: Clinical Frailty Scale.

**Table 2:** Inclusion and Exclusion Criteria.

Inclusion Criteria	Exclusion Criteria
Age between 18 and 80 years Diagnosis of liver cirrhosis (based on clinical, laboratory and imaging findings) Absence of any active condition that could affect frailty assessment	• Presence of any of the following: ○ Active malignancy ○ Central nervous system disease ○ Acute kidney injury • Any orthopedic condition limiting mobility

**Table 3:** Fried Frailty Index and Cut-off Values.

Criterion	Women	Men
Weight loss	Unintentional weight loss >4.5 kg or >5% over the past year	
Walking time (4.5 m)	Height ≤159 cm: ≥7 s Height >159 cm: ≥6 s	Height ≤173 cm: ≥7 s Height >173 cm: ≥6 s
Grip strength	BMI ≤23: ≤17 kg BMI 23.1–26: ≤17.3 kg BMI 26.1–29: ≤18 kg BMI >29: ≤21 kg	BMI ≤24: ≤29 kg BMI 24.1–26: ≤30 kg BMI 26.1–28: ≤30 kg BMI >28: ≤32 kg
Physical activity	<270 kcal/week (≈2 h of walking)	<383 kcal/week (≈2.5 h of walking)
Exhaustion	Positive response (≥3–4 days/week) to either of the following CES-D items: “I felt that everything I did was an effort.” or “I could not get going.”	

BMI: Body Mass Index, CES-D: Center for Epidemiologic Studies Depression Scale.

**Table 4:** Baseline Demographic and Clinical Characteristics of the Patients.

Characteristics	Patient Groups		*p*-Value
	Frail N = 55	Fit/Prefrail N = 79	
Age, years	67 (61–73)	59 (53–67)	<0.001
Sex			0.347
Male	24 (44%)	41 (52%)	
Female	31 (56%)	38 (48%)	
BMI, kg/m^2^	30.4 (27.2–34.6)	29.6 (25.4–32.7)	0.189
Comorbidities			
Hypertension	30 (55%)	28 (35%)	0.028
Diabetes mellitus	38 (69%)	40 (51%)	0.033
Coronary artery disease	17 (31%)	5 (6%)	<0.001
Etiology of cirrhosis			0.129
Chronic viral hepatitis	7 (13%)	20 (25%)	
Alcohol	4 (7%)	4 (5%)	
NASH	23 (42%)	27 (34%)	
Autoimmune liver disease	3 (5%)	11 (14%)	
Cryptogenic and other *	18 (33%)	17 (22%)	
MELD score	11.0 (9.0–16.0)	9.0 (7.0–11.0)	<0.001
CTP score	7.0 (6.0–7.5)	5.0 (5.0–6.0)	<0.001
CTP classification			<0.001
A	21 (38%)	59 (75%)	
B	30 (55%)	17 (22%)	
C	4 (7%)	3 (4%)	
Ascites			<0.001
None	16 (29%)	59 (75%)	
Mild/controlled	38 (69%)	19 (24%)	
Moderate/severe	1 (2%)	1 (1%)	
Varices	38 (69%)	52 (66%)	0.692
History of variceal bleeding	12 (22%)	18 (23%)	0.895
Beta-blocker use	44 (80%)	58 (73%)	0.257
Furosemide use	21 (38%)	17 (22%)	0.035
Spironolactone use	29 (53%)	17 (22%)	<0.001
Rifaximin use	9 (16%)	4 (5%)	0.030
L-ornithine L-aspartate use	17 (31%)	19 (24%)	0.378

* Other causes: cardiac cirrhosis, granulomatous liver disease (n = 3). BMI: Body mass index; MELD: Model for End-stage Liver Disease; CTP: Child–Turcotte–Pugh; NASH: Non-alcoholic steatohepatitis.

**Table 5:** Complications and Clinical Outcomes of the Patients.

Complications and Outcomes	Frail N = 55	Fit/Prefrail, N = 79	*p*-Value
Ascites development	19 (35%)	7 (8.9%)	**<0.001**
Hepatic encephalopathy	27 (49%)	4 (5.1%)	**<0.001**
Variceal bleeding	8 (15%)	4 (5.1%)	0.071
Hepatorenal syndrome	13 (24%)	1 (1.3%)	**<0.001**
Spontaneous bacterial peritonitis	5 (9.1%)	0 (0%)	**0.010**
Hepatocellular carcinoma	2 (3.6%)	2 (2.5%)	>0.999
Hospitalization	30 (55%)	13 (16%)	**<0.001**
Mortality	12 (22%)	3 (3.8%)	**<0.001**
Composite outcome	40 (73%)	17 (22%)	**<0.001**

Bold formatting note significant results.

## Data Availability

Due to the sensitive nature of the data, it is not publicly accessible; however, it may be provided by the corresponding author upon reasonable request.
